# Five questions that need answering when considering the design of clinical trials

**DOI:** 10.1186/1745-6215-15-286

**Published:** 2014-07-16

**Authors:** Timothy Clark, Hugh Davies, Ulrich Mansmann

**Affiliations:** 1 Institut für Medizinische Informationsverarbeitung, Biometrie und Epidemiologie (IBE), Faculty of Medicine, Ludwig-Maximilians University, Munich, Germany; 2 Health Research Authority, Skipton House, London SE1 6LH, UK

**Keywords:** Research protocol, Research question, Ethical research, Systematic review, Sample size, Design assumptions, Justification, Monitoring, Study registration, Publication

## Abstract

Evidence suggests that research protocols often lack important information on study design, which hinders external review. The study protocol should provide an adequate explanation for why the proposed study methodology is appropriate for the question posed, why the study design is likely to answer the research question, and why it is the best approach. It is especially important that researchers explain why the treatment difference sought is worthwhile to patients, and they should reference consultations with the public and patient groups and existing literature. Moreover, the study design should be underpinned by a systematic review of the existing evidence, which should be included in the research protocol. The Health Research Authority in collaboration with partners has published guidance entitled ‘Specific questions that need answering when considering the design of clinical trials’. The guidance will help those designing research and those reviewing it to address key issues.

## Background

Safe advances in health care require that we are able to appraise critically both the proposed design and the subsequent results of clinical studies. Proper design is crucial to a study’s success and ethical acceptability, and sample size is a major component of design [1]. Our recent review of sample size determinations in research protocols for randomised clinical trials submitted to United Kingdom research ethics committees (RECs) found that most did not contain sufficient information to allow the sample size to be reproduced, or the plausibility of the assumed treatment effect or variability used in the calculation to be assessed [2,3]. Overall, only 55 out of 446 (12%) protocols reported both the data on which the treatment effect sought was based and its clinical importance; 135 (30%) protocols reported the data only and 256 (57%) reported neither. Limited information on the nature of the data underpinning the treatment effect was usually given, and just 13 (3%) protocols gave a reasoned explanation why the value chosen was plausible for the planned study.

Our research raised the question of whether randomised controlled trials are appropriately designed to answer the research question posed. We concluded that greater transparency in the reporting of the determination of the sample size and more focus on study design during the ethical review process were needed to allow deficiencies to be resolved early, before the trial begins. The Health Research Authority (HRA) in collaboration with the University of Munich and other partners has now developed necessary guidance entitled ‘Specific questions that need answering when considering the design of clinical trials’. This guidance, which lays out questions that researchers, sponsors, peer reviewers and ethics committees should ask when planning or reviewing clinical studies, has been published on the HRA website [4]. The complete guidance is provided in Additional file 1.

## Main text

### The HRA Guidance

This HRA guidance poses five questions [4]:

1) Is there a clear research question?

2) Will the proposed study design answer the research question?

3) Are the assumptions used in the sample size calculation appropriate?

4) How will safety and efficacy be monitored during the trial?

5) How will the trial be registered and subsequently published?

#### Document structure

The HRA guidance is separated into four layers, providing increasing detail, as required. Layer I sets out in tabular form the questions and considerations that researchers, sponsors, peer reviewers and RECs should ask. By clicking over a question or specific words or phrases in the table in Layer I the reader can navigate to a more detailed discussion of the topic in Layer II. From Layer II the reader can navigate to more comprehensive explanations of individual components of the sample size in Layer III. Finally, Layer IV provides explanatory notes on some underlying statistical principles.

### The five questions

The HRA guidance is built on the following core concepts: that sound planning is critical to the outcome of an interventional clinical trial and its ethical acceptability (bad science is bad ethics); all trials must be registered; and the results should be published [5,6]. A researcher should identify the primary research question to be answered; explain why it is important to patients and health-care practitioners; show that the study design is appropriate to answer the question posed, in particular that the sample size is likely to be adequate to meet the pre-specified aims of the study; and describe the plan to disseminate the results of the study.

#### ***Is there a clear research question?***

The definition of the research question is key to research design. All research must have a primary question, clearly stated in advance, and founded on a systematic review of what is already known [5,7-9]. Researchers who plan studies without reviewing what has been done, risk performing research for which the answer is already known or exposing participants to ineffective or an inferior treatment [8-10].

The planning of a clinical trial depends on the primary question, and researchers should clearly and simply explain in the study protocol what the trial is aiming to show, why it is worth asking and, through consultation with public and patient groups, why this is worthwhile to patients. The primary question should be consistently stated throughout the study protocol. Population, intervention, comparator and outcome (PICO) is one useful way of formulating a research question [11].

#### ***Will the proposed design answer the research question?***

The research protocol should explain how the proposed study methodology is appropriate for the question posed, demonstrate that the design is likely to answer the research question, and why it is the best approach. The design should be underpinned by a systematic review of the existing evidence, which should be reported in the protocol [8,9]. Absence of a systematic review raises the question: what is the design based on?

Unfortunately, our and other research has found that study protocols often lack important information on study design, which hinders external review [2,3,7,12]. The HRA guidance therefore encourages researchers to explain:

• how the proposed research method is appropriate for the question posed

• the reasoning behind the choice of any treatment difference sought, as well as the other parameters used in the determination of the sample size

• how the relevant successes and failures of previous studies have been taken into account in the design of the planned trial

• the reason for the choice of comparators

• the randomisation and blinding methods

• the suitability of the statistical tests

• how the sample to be studied is representative and thus, generalisable to the wider group of patients

#### ***Are the assumptions used in the sample size calculation appropriate?***

Researchers should provide all the information needed to allow an independent reviewer to both reproduce the target sample size and understand the rationale for the assumptions used in the calculation. The HRA guidance provides a checklist of the information that should be reported in the study protocol for the sample size determination. Each item is linked to more detailed explanations. Specific guidance is given on reporting sample sizes based on confidence intervals, group sequential, factorial, cluster and time-to-event studies.

The sample size determination checklist requests that researchers:

• Explain what the study is aiming to show

• Describe the design of the study

• State clearly the primary outcome measure

• State the test procedure on which the sample size is based

• State the allocation ratio

• State and justify the difference sought, if the study is aiming to show superiority

• State and justify the acceptance margin, if the study is aiming to demonstrate

• non-inferiority or equivalence

• Report all parameters used in the sample size calculation

• Explain the rationale for the parameters used in the sample size calculation

• Describe any procedures to re-estimate the sample size during the study

• Report the Type I error

• Report the Type II error

• Describe any adjustments for multiple testing (multiplicity), if the study has multiple endpoints, interim analyses, or multiple arms

• State the number of patients or events required for the analysis

• Explain the allowance (if any) for dropouts

• State the total number of patients to be enrolled

Particular emphasis is placed on the need to provide a reasoned explanation of why the treatment difference and other design assumptions are plausible for the planned study, taking into account all existing data. The importance of the difference sought, i.e. why it is worthwhile to patients, should also be explained. The justification could include reference to consultations with the public and patient groups, existing literature or published studies in which the minimum clinically important difference has been empirically determined.

Researchers should be rigorous in the determination of design assumptions [1-3]. Sample size re-estimation should be considered if there is a high degree of uncertainty [13]. Manipulating the sample size calculation, sometimes called the sample size ‘game’ or ‘samba’, to produce the desired statistical power by inappropriately overestimating the treatment effect (known as ‘optimism bias’ – the unwarranted belief in the efficacy of new therapies) or underestimating the variability leads to ‘underbuilt’ studies with insufficient power and inconclusive results [14,15]. Such studies are not ethical and waste valuable resources [16-18].

Sample size determinations must be realistic [19]. If the sample size required to detect the treatment difference of interest is unfeasible, then this should be explained in the research protocol. A well-designed and implemented trial, even one with lower power (and precision), will still yield unbiased results, which can be combined with similar unbiased trials in a meta-analysis [14].

#### ***How will safety and efficacy be monitored during the trial?***

All studies should be monitored for protocol compliance, adverse effects, patient recruitment, etc. If treatment is of long duration then accumulating efficacy data should be monitored for overwhelming evidence of efficacy or harm. No study should continue to recruit patients once the main comparisons have revealed clear-cut differences [5].

The repeated significance testing of accumulating data does have statistical implications [5]. Thus, the research protocol should describe how multiple testing has been accounted for in the sample size determination [2,3,7]. If there is a great deal of uncertainty surrounding the design assumptions then researchers are asked to consider re-estimating the sample size during the course of the study.

#### ***How will the trial be registered and subsequently published?***

For clinical trials, HRA has now established that a favourable REC opinion is contingent upon trial registration in publicly accessible databases, unless acceptable reasons are provided for not doing so. The REC also needs to be assured that results will be placed in the public domain. Researchers are expected to:

• publish their results in full and in a reasonable timescale, even when they do not match expectations

• follow reporting guidelines for clinical trials (e.g., CONSORT [20])

• discuss their findings in the context of an updated systematic review of relevant research

• provide their results to others doing systematic reviews of similar topics

## Conclusion

It is axiomatic that bad science is bad ethics. Poor research design puts us all at risk. Participants may be exposed to an inferior treatment, or enrolled in trials that provide no useful information on which to build health care. Present and future patients may receive ineffective treatment. Our collaboration provided evidence that we need to address the design of clinical trials; our guidance will help those designing such research and those reviewing it to address key issues, facilitating ethical research that will underpin and improve health care, a key role for HRA.

## Abbreviations

CONSORT: CONsolidated Standards of Reporting Trials; HRA: Health Research Authority; PICO: population, intervention, comparator, outcome; REC: research ethics committee.

## Competing interests

All authors have completed the International Committee of Medical Journal Editors uniform disclosure form [21] (available on request from the corresponding author) and declare the following. HD and UM received no support from any organisation for the submitted work. TC received support for travel from the HRA and has worked as a consultant for ICON plc in the previous 5 years. The authors have no other relationships or activities that could appear to have influenced the submitted work.

## Authors’ contributions

TC drafted the manuscript. HD and UM undertook a critical review of the manuscript. HD is the guarantor. All authors read and approved the final manuscript.

## Supplementary Material

Additional file 1 This is the published HRA guidance entitled ‘Specific questions that need answering when considering the design of clinical trials’. Click here for file
