# Supplementary material for: Five questions that need answering when considering the design of clinical trials
Source: Trials. 2014 Jul 16;15:286. doi: 10.1186/1745-6215-15-286 (PMC4223595; doi:10.1186/1745-6215-15-286)
Supplement: Additional file 1 — This is the published HRA guidance entitled ‘Specific questions that need answering when considering the design of clinical trials’. [file 1745-6215-15-286-S1.pdf]

NHS
Health Research Authority

Guidance

Specific questions that need answering when considering the design of clinical trials

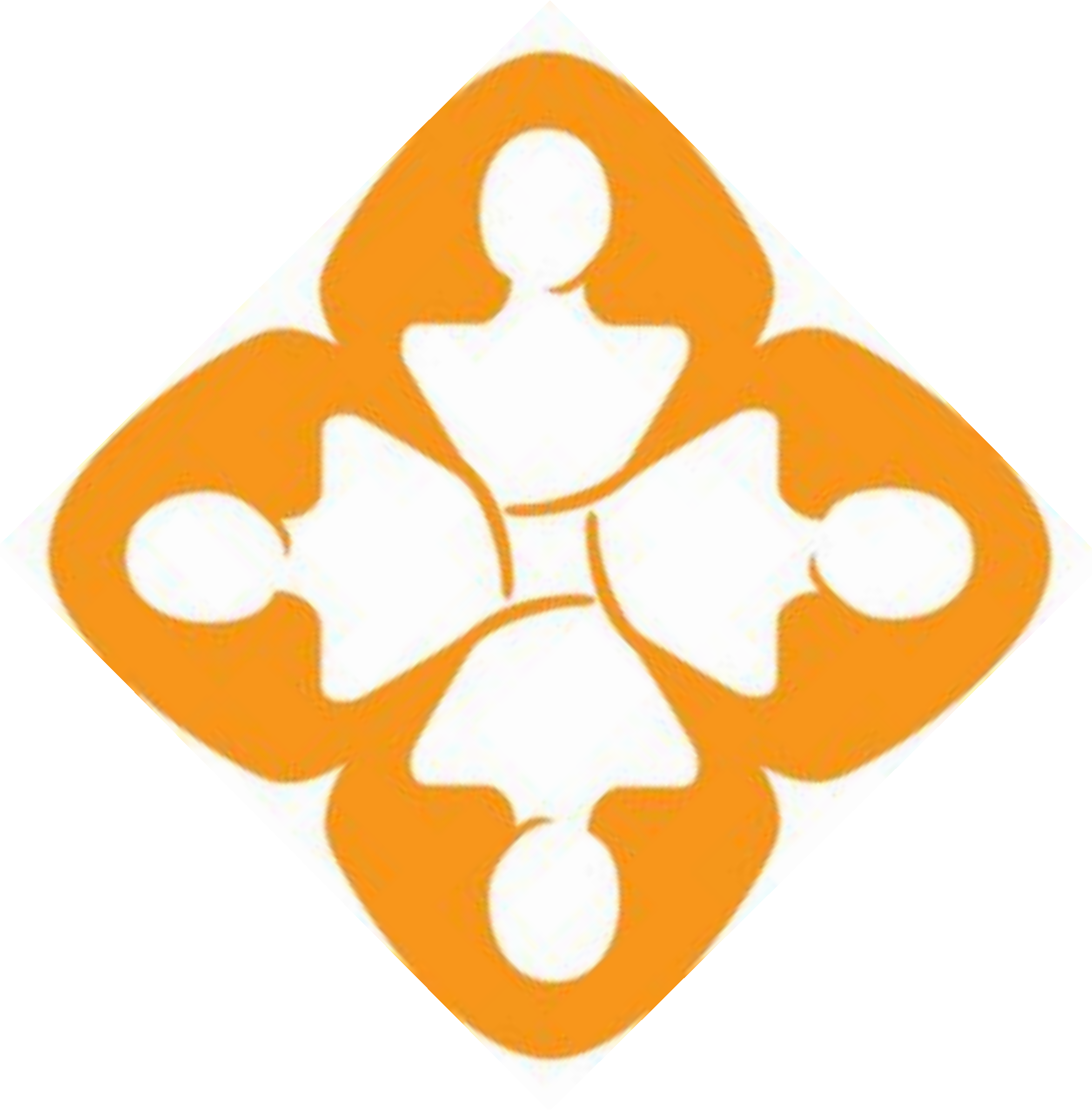

Information can be reproduced only with permission.

Evidence indicates that the quality of clinical trial design and research protocols is not as good as it could be[1-5]. This guidance is our contribution to making it better. It lays out questions researchers, sponsors, peer reviewers, and Research Ethics Committees (RECs) should ask when considering their study design.

The structure of this document and how to use it.

This document is “layered”, providing increasing detail if needed. It is not designed to be read from beginning to end. The reader should first consider the questions in **Layer I** and then **click** over the term or phrase if more information is required.

> **Layer I** sets out in tabular form the “**Questions**” and “**Considerations**” that researchers, sponsors, peer reviewers, and RECs should ask
>
> ⇓
>
> By **clicking** over the question or specific words or phrases in the table in **Layer I** the reader will be able to navigate to a more detailed discussion in **Layer II**
>
> ⇓
>
> By **clicking** over specific terms or phrases in **Layer II** the reader will be able to navigate to more detailed explanations of individual components of the sample size in **Layer III**
>
> ⇓
>
> By **clicking** over specific terms or phrases in **Layer III** the reader will be able to navigate to explanatory notes on some underlying statistical principles in **Layer IV**
>
> ⇓
>
> **Click** on the link at the end of each section to get back to the first layer.

Provenance

In developing this guidance we have combined material initially developed for National Research Ethics Service training workshops, papers arising from a collaboration between the Health Research Authority (HRA) and the University of Munich, Institute for Medical Informatics, Biometry and Epidemiology and advice provided by reviewers from the National Institute for Health Research’s research programmes including the Health Services & Delivery Research Programme (NIHR HS&DR programme) which funds research focusing on improving outcomes for health and social care[1, 6].

We are particularly grateful to Tim Clark who led the collaboration and drafted the subsequent guidance. We are also grateful to Charles Beck, Steph Garfield-Birkbeck, Sue Bourne, Iain Chalmers, Clive Collett, Gary Collins, David Dickinson, Ron Driver, Ulrich Mansmann, Victoria Owen, Margaret Stoddart, Matthew Sydes, Nick Taub, and Gordon Taylor who contributed to our initial training days and an HRA workshop on this topic, and commented on earlier drafts of this guidance. David Dickinson also helped with the design and editing of this guidance.

Questions	Considerations
**1. Is there a clear research question?**<br>Is the research question clearly and consistently stated?<br>Is there a satisfactory review of current knowledge?<br>Is this question of potential importance to patients and health care practitioners?	*A clear research question is the cornerstone of good research practice.*<br>*Any project should build on a review of current knowledge. Replication to check the validity of previous research is justified, but unnecessary duplication is unethical.*<br>*Researchers should explain why the question is worth asking.*
**2. Will the proposed study design answer the research question?**<br>What is the primary outcome measure of the research?<br>What is the treatment difference?	*Researchers should be able to explain how the proposed research method is appropriate for the question posed, demonstrate that the design will answer the research question, and why it is the best approach.*<br>*The primary outcome measure should be a clear, quantitative measure of effect which, along with the time it will be measured, should be plainly and consistently described in the study protocol.*<br>*Researchers should present the evidence that any treatment difference they are seeking to detect is clinically important to patients and realistic.*
**3. Are the assumptions used in the sample size calculation appropriate?**<br>Is there evidence and reasoning behind the design assumptions used in the calculations?<br>Can the calculation be reproduced?	*Researchers must report all the information needed to allow any reader to understand the rationale for the assumptions that have been made and reproduce the sample size.*<br>*The sample size should always be justified.*
**4. How will safety and efficacy be monitored during the trial?**<br>Is the safety of participants adequately protected?	*Researchers have a responsibility to the study participants to monitor safety and clinical benefit during the trial.*<br>*The sample size normally needs adjustment for planned interim analyses.*
**5. How will the trial be registered and subsequently published?**<br>Are there plans to place the project in the public domain by registration and publication?<br>How and when will these results be shared with the participants?	*Ethical research is open. Trials should be registered and results published. The HRA has made registration a condition of the favourable REC opinion and is leading further work.*

1. Research Question

Definition of the research question is key to research design. It should be:

- ☑ Clearly and consistently stated
- ☑ Worth asking
- ☑ Based on systematic review of relevant exiting evidence

All research must have a primary question, clearly stated in advance. The planning of a clinical trial depends on this question and researchers should explain clearly and simply in the study protocol what the trial is aiming to show, why it is worth asking and, through consultation with the public and patient groups, why this is worthwhile to patients.

Researchers should ensure that the primary question is consistently stated throughout the study protocol.

Underpinning this, researchers should conduct a systematic review of the relevant existing evidence before starting the study and report this in the protocol. Absence of a systematic review raises the question: what is the design based on[7-9]?

Back to table

2. Study Design

Here we are asking, "Will the research, as designed, answer the research question?"

Researchers should be able to:

- ☑ clearly state the research question and the primary outcome measure
- ☑ explain how the research method proposed is appropriate for the question posed, and why it is the best approach
- ☑ provide sound reasoning behind the choice of any treatment difference sought, as well as the other parameters used in the determination of the sample size
- ☑ describe in the study protocol how the relevant successes and failures of previous studies have been taken into account in the design of the planned trial[8-9]
- ☑ justify the comparators
- ☑ explain the blinding and randomisation methods, and why the statistical tests applied are suitable
- ☑ show that the sample studied is representative and thus, generalizable to the wider group of patients. Inclusion and exclusion criteria need to be adequately described

Back to table

2.1 The primary outcome measure

A single or composite primary outcome measure (also called the primary endpoint) should be identified to address the primary question. Composite outcomes combine multiple outcome measures within a single variable. The primary outcome measure should be a clear, unarguable, quantitative measure of effect. The outcome measure and the time at which it will be measured should be clearly and consistently stated in the study protocol.

> ***Example**: 'Patients alive 6 months after start of treatment' or 'Mean change from baseline to Week 52 in HbA1c level'.*

In any clinical study, it is usual to specify one primary outcome measure, considered more important than others, and on which the sample size calculation is normally based.

If the sample size is not based on the primary outcome measure, but on a secondary efficacy or safety measure, or sub-analyses, this needs to be clearly explained in the sample size determination.

There can be any number of secondary measures, although they should all be relevant to the declared aims of the study.

Back to table

2.2 Difference sought or acceptance margin

In a superiority trial, researchers should clearly describe any treatment difference they are seeking to detect and present evidence that it is clinically important to patients and realistic. This is often referred to as the minimum clinically important difference or the difference one would not like to miss. A treatment which reduces everyone's systolic blood pressure by 2mm of mercury may be genuinely effective, but the effect would not form the basis of a routine intervention.

For equivalence or non-inferiority designs, researchers should demonstrate that the acceptance margin represents the largest difference that is clinically acceptable to patients.

Researchers should therefore provide sound reasoning behind the choice of treatment difference or acceptance margin used in the determination of the sample size.

Underpinning this, researchers should conduct a systematic review of the relevant existing evidence before starting the study and report this in the protocol. Absence of a systematic review raises the question: what is the design based on[7-9]?

Back to table

2.3 Types of clinical trials

Design will depend on the research question and what the study is designed to show.

Comparative trials are usually designed to show:

- **Superiority**: treatment is superior to the comparator
- **Non-inferiority**: treatment is ‘at least as good’ or ‘not worse than’ the comparator
- **Equivalence**: treatment is ‘not worse than’ and ‘not better than’ the comparator

Common designs include parallel group, crossover, factorial, cluster and group sequential.

Parallel group design: each group of participants receives only one of the study treatments.

Cross-over design: participants are given all the study treatments sequentially in time. The order in which the participants receive each treatment is determined at random.

Factorial design: two or more treatments are evaluated separately and in combination against a control. For instance, the ISIS-2 study employed a 2x2 factorial design to assess the separate and combined effect of intravenous (IV) streptokinase (SK) and of aspirin in decreasing the risk of vascular mortality in patients with suspected acute Myocardial Infarction[10]. Patients were randomised to receive an IV infusion of SK plus aspirin tablets, SK plus matching placebo tablets, aspirin plus matching placebo IV infusion, or placebo IV infusion and placebo tablets.

Cluster randomised: the treatment is randomised to groups of participants (e.g. families, GP surgeries, schools, and towns) rather than individual participants.

Groups sequential: outcomes are assessed in a group and sequential manner. A decision to stop the trial prematurely can be taken due to overwhelming evidence of efficacy or serious safety problems or futility (i.e. there is little chance of a positive conclusion). Group sequential designs involve multiple looks at accumulating data, which gives rise to multiplicity.

Multiple-armed design: study with more than two arms. Examples of multiple-armed studies would include two or more different test treatments versus control, or a test treatment versus an active and a placebo control.

Back to table

2.4 Study comparators

In a comparative trial, the test treatment is compared against a control, which could be placebo or the standard treatment. The placebo or standard treatment is called the comparator. Researchers need to justify the comparators (not just experimental interventions), particularly if a study involves withholding or delaying standard of care.

Back to table

Please note: Peer reviewers for the NIHR's research programmes are asked to consider the soundness of the study design during the appraisal process[6]. For example:

Proposed research methods, recruitment and scientific quality:

- Are the research methods robust?
- Will the research methods suggested provide answers to the research question(s)?
- Are the plans for data collection and analysis sufficiently detailed?
- Are any assumptions made? Are they reasonable?
- Is the study design appropriate?
- Are the outcomes and duration of follow up appropriate?
- Are the research participants and the methods of identifying them relevant and sufficient?
- Are the interventions adequately described and appropriate?
- Are the effects, measures or information sought appropriate and relevant to the study's purpose?
- Where appropriate, is there a clear explanation and justification for the sample size and/or estimated recruitment rates?
- Where appropriate, is there a realistic chance of recruiting the required number of participants within the time stated? Is the sample size based on a believable effect size?
- Are you aware of any overlap with research currently underway?

Back to table

3. The Sample Size Determination

The following is a checklist of the information on the sample size determination that should be reported in the study protocol. Researchers should make every effort to report all the information needed to allow an independent reviewer to reproduce the sample size and understand the rationale for the assumptions used in the calculation.

Caution: All study protocols must provide some form of sample size justification.

Recommendation: It is important to involve a statistician from the beginning to ensure that the sample size and analysis plan is appropriate for the study.

Explain what the study is aiming to show
Describe the design of the study
State clearly the primary outcome measure
State the test procedure on which the sample size is based
State the allocation ratio
Superiority design: state and justify the difference sought
In non-inferiority or equivalence designs state and justify the acceptance margin for the study
Report all other parameters used in the sample size calculation
Report a study with time-to-event outcome measures
Report a group sequential design
Report a factorial design
Report a cluster design
Sample size based on confidence intervals or feasibility
Explain the rationale for the parameters used in the sample size calculation
Sample size re estimation
Report the Type I error
Report the Type II error
Adjustment for multiplicity
State the number of patients or events required for the analysis
Explain the allowance (if any) for drop-outs
Record the total number of patients to be enrolled

Back to table

Peer reviewers for the NIHR's research programmes are asked to consider the appropriateness of the sample size and any assumptions made during the appraisal process[6]. For example:

Proposed research methods, recruitment and scientific quality

- Are any assumptions made? Are they reasonable?
- Is the study design appropriate?
- Are the outcomes and duration of follow up appropriate?
- Are the research participants and the methods of identifying them relevant and sufficient?
- Are the effects, measures or information sought appropriate and relevant to the study's purpose?
- Where appropriate, is there a clear explanation and justification for the sample size and/or estimated recruitment rates?
- Where appropriate, is there a realistic chance of recruiting the required number of participants within the time stated? Is the sample size based on a believable effect size?

Back to table

4. Monitoring safety and efficacy during the clinical study

All studies should be monitored for protocol compliance, adverse effects, patient recruitment, etc. If treatment is of long duration then accumulating efficacy data should be monitored for overwhelming evidence of efficacy or harm. No study should continue to randomise patients once the main comparisons have revealed clear-cut differences.

The repeated significance testing of accumulating data does have statistical implications. Thus, the research protocol should describe how multiple testing has been accounted for in the sample size determination.

There my also be a need to revisit the sample size during the course of the clinical study if there is uncertainty about the assumptions used in the sample size calculation.

Please note: The DAMOCLES Study Group has proposed a charter for clinical trial data monitoring committees, and the European Medicines Agency (EMA) and the Food and Drug Administration (FDA) have both published guidance for clinical trial sponsors[11-14].

Back to table

5. Registering and Publishing the Results

The REC needs to be assured that: (1) access to results will not be unfairly restricted; (2) results will not be kept in the hands of those with conflicting interests; and (3) results will be made publically available.

For clinical trials, favourable REC opinion is contingent upon trial registration in publically accessible databases.

Researchers are expected to:

- publish their results in full and in a reasonable timescale, even when they do not match expectations
- follow reporting guidelines for clinical trials (e.g. CONSORT)[15]
- discuss their findings in the context of an updated systematic review of relevant research
- provide their results to others doing systematic reviews of similar topics

Back to table

Components of the Sample Size Determination

State the test procedure on which the sample size is based

Researchers need to specify the statistical test procedure used to calculate the sample size, e.g. two sample (unpaired) t-test, paired t-test, chi-squared tests, Fisher's Exact test, Mantel-Hansel test, log-rank test.

Caution: the way the sample size is calculated is determined by the way the data will be analysed. Researchers should begin by thinking of the analysis that will ultimately be performed on the primary outcome measure to ensure that the sample size is calculated appropriately.

> ***Example for two sample t-test***: *a sample size of 166 in each group (1:1) will have 90% power to detect a difference in means of 0.5% in change from baseline to Week 52 in HbA1c level, assuming that the common standard deviation is 1.4%* ***using a two sample t-test with a 0.05 two-sided significance level****. Allowing for 10% losses to follow-up (drop-out), at least 185 patients per group should be enrolled.*

Caution: The t-test assumes that the distribution of the variables is approximately normal and that the variances are equal.

If the *chi-squared test* has been used, specify whether the variance is *pooled* or *unpooled*. This refers to the way in which the standard error is estimated:

- Pooled = two proportions are averaged, and only one proportion is used to estimate the standard error
- Unpooled = two proportions used separately

If the **chi-squared with continuity correction** has been used, then this should be specified.

Caution: The sample size must be large enough so that the expected frequency in each cell is greater than or equal to 5.

If the sample size is based on more complex methods of analysis such as *Analysis of Covariance (ANCOVA)* or *logistic regression*, provide one or more of the following:

- Full details of all components of the sample size calculation
- The precise formula used or a reference to the relevant publication
- The sample size software used, e.g. PASS 12 / Logistic Regression. Wherever possible a copy of the report from the statistical software should be provided in an appendix to the study protocol.

Back to table

State the allocation ratio

The allocation ratio is the ratio of the number of participants allocated to different treatment groups. For example, equal numbers in two groups gives a ratio of 1:1; twice as many patients in the treatment group as in the control group is expressed as a ratio of 2:1.

Back to table

Superiority design: state and justify the difference sought

In a superiority trial the objective is to demonstrate that the test treatment is better than the comparator. The research protocol should clearly describe the treatment difference sought and present evidence that it is clinically important to patients and realistic.

> ***Example***: *a sample size of 166 in each group (1:1) will have 90% power* ***to detect a difference in means of 0.5% in change from baseline to Week 52 in HbA1c level****, assuming that the common standard deviation is 1.4% using a two sample t-test with a 0.05 two-sided significance level. Allowing for 10% loss to follow-up (drop-out), at least 185 patients per group should be enrolled.*

Back to table

Non-inferiority or equivalence design: state and justify the acceptance margin for the study

In a **non-inferiority study,** the objective is to show that the test treatment is ***either*** "no worse than" ***or*** "no better than" the comparator.

Researchers need to clearly report the **acceptance margin**, which represents **a clinically unimportant difference**. That means a difference that is clinically acceptable such that a larger difference than this would matter in clinical practice[16].

The **true or expected difference between treatments** should also be reported. This is usually zero (absolute difference) or 1.00 (ratio).

> ***Example****: a sample size of 253 patients in each group will give 80% probability of demonstrating that the test is non-inferior to the comparator treatment (****one-sided 97.5% confidence interval for the difference in mean change from baseline to Week 8 in blood pressure, μ_T - μ_R, is entirely to the right of the -1.5 mmHg non-inferiority margin****), assuming that the* ***expected difference in means is 0.000*** *and the common standard deviation is 6.000. Allowing for 10% losses to follow-up (drop-out), at least 282 patients per group should be enrolled.*

In an equivalence study, the objective is to show that test treatment is ***both*** "no worse than" ***and*** "no better than" the comparator.

> ***Example***: *a sample size of 275 patients per treatment group will give 80% probability of demonstrating that test is equivalent to comparator treatment (**two-sided 95% confidence interval for the difference in mean change from baseline to Week 8 in blood pressure, μ_T - μ_R, completely contained between the equivalence region [-1.5 mmHg, 1.5 mmHg]**), assuming that the **expected difference in means is 0.000**, the common standard deviation is 6.000 and that each test is made at the 5.0% level. Allowing for 10% losses to follow-up (dropout), at least 306 patients per group should be enrolled.*

Researchers need to report the **acceptance margin**, which represents **a clinically unimportant difference**. That means a difference that is clinically acceptable such that a larger difference than this would matter in clinical practice[16].

The **true or expected difference between treatments** should also be reported. This is usually zero (absolute difference) or 1.00 (ratio).

Back to table

Report all other parameters used in the sample size calculation

Depending on the design of the study, researchers will also need to report one or more of the following parameters in addition to the treatment difference or acceptance margin:

Continuous data: variance in each treatment group. Variance often assumed to be equal in each group.

Binary data: proportion of participants in the control group with success / failure; alive / dead; with / without symptoms.

Survival data (time-to-event): median survival times in each group; hazard rates; hazard ratio.

> ***Example for continuous data***: *a sample size of 166 in each group (1:1) will have 90% power to detect a difference in means of 0.5% in change from baseline to Week 52 in HbA1c level, **assuming that the common standard deviation is 1.4%** using a two sample t-test with a 0.05 two-sided significance level. Allowing for 10% losses to follow-up (drop-out), at least 185 patients per group will be enrolled.*

If using more complex methods of analysis such as *ANCOVA* or *logistic regression*, researchers should report all the parameters used in the calculation (e.g. number of covariates and the average R-squared value (coefficient of determination) between the response and the covariates).

Back to table

Reporting a study with time-to-event outcome measures

For these studies, the number of events is important and a sufficient number of participants should be recruited to ensure a sufficient number of events. The number will depend on the **accrual period** (i.e. the period over which participants are recruited and enrolled) and the **overall study duration** (i.e. accrual period + follow-up period).

Recommendation: The planned accrual period and follow-up period should be reported in the study protocol, with the methodology to estimate the sample size. Be sure to provide enough information to allow a reviewer to easily understand how the calculation was performed. Wherever possible, a copy of the report from the statistical software should be provided in an appendix to the study protocol.

Back to table

Reporting a group sequential design

If the study has a group sequential design then the number of interim looks, stopping rules, and the survival time assumption should be reported. Group sequential designs involve multiple looks at accumulating data, which gives rise to multiplicity.

Number of Looks. This is the number of interim analyses (including the final analysis).

Stopping rules for interim analysis: if there are stopping rules for efficacy, safety, and / or futility, then these should be reported clearly.

Stopping rules for efficacy: common approaches include the O'Brien and Fleming method, and the Lan and DeMets alpha spending function.

Survival Time Assumption: have exponential survival times or proportional hazards only been assumed?

Back to table

Reporting a factorial design

Caution: factorial design assumes that the effects of the two treatments are independent, i.e. that the effect of treatment A does not differ depending on whether participants also receive treatment B. In other words, **there is no interaction** between the treatments

Recommendation: If the study has a factorial design, researchers should carefully consider why it is reasonable to assume that there is no interaction between the treatments. This should be reported in the sample size determination

Caution: With no increase in sample size, the interaction needs to be twice as large as the main effect in order to be detected with the same statistical power.

Back to table

Reporting a cluster design

Researchers should state clearly whether they have assumed that cluster sizes are all the same or are likely to vary.

If a **variable cluster size** has been assumed, researchers should explain how this has been accounted for in the sample size estimation.

Researchers should report the estimated intra-cluster correlation coefficient (ICC) and how this was estimated.

Back to table

Sample size based on confidence intervals or feasibility

If the study is not designed to formally investigate a hypothesis but to obtain a point estimate of the treatment effect and a confidence interval, then this should be stated as the study's objective and as the basis of the sample size of the study. The population variance and the required width of the confidence interval should be reported in the sample size determination.

Alternatively, if the sample size is based on feasibility, researchers should report the anticipated precision of any treatment effect, if say a 95% confidence interval is used, or what difference can be detected with 80% or 90% power.

Back to table

Rationale for the parameters used in the sample size calculation

The research protocol should include **a concise summary** of how the assumptions used in the sample size calculation were chosen and why they are considered plausible for the planned study.

Researchers should show the reviewers that they have **solid reasoning** behind their calculations. Researchers are encouraged to **include tables and graphs** of the data on which the parameters are based, as these can be very helpful.

The clinical importance of the difference sought, i.e. why it is worthwhile to patients, should be explained. Just saying that the "treatment difference is clinically important" without further explanation is meaningless. The justification could include reference to consultations with the public and patient groups, existing literature, and / or published studies in which the minimum clinically important difference has been empirically determined.

Caution: The sample size calculation is only as accurate as the data/estimates on which it is based.

If researchers are **too optimistic about the size of the expected treatment difference**, the sample size will be too small, and the study may not have sufficient power to detect the minimum clinically important difference – in which case **it will be inconclusive**.

Good information is required for the **variability or median survival time**. Again, if researchers are **too optimistic** in their choice (for example, if they underestimate the variability of success rates in treated patients) the sample sizes will be too small, and **the study will be severely underpowered**.

The **design assumptions** for the study should take account of data from previous studies and any systematic review of existing relevant evidence.

Choose previous studies that are as similar as possible to the planned trial.

Recommendation: researchers are encouraged to consider the following aspects:

- Is the design of the previous studies similar to the one being planned?
- Are the outcome measures similar in terms of how they are defined and the time point at which they are measured?
- Is the statistical analysis in the planned trial the same as that undertaken in the previous studies?
- Are the demographic and disease characteristics of the patient populations similar?
- If the previous studies were performed with the treatment that will be investigated in the planned trial, was the dose, duration of treatment, length of follow-up similar?
- Are there data from small early phase studies or open-label studies? These study designs can often over-estimate treatment effects.
- How has medical treatment changed since the previous studies were conducted?
- Is standard medical care likely to change during the course of the study?
- Is the planned study going to be conducted in the same countries and geographical regions? Different countries may have different types of care (e.g. concomitant medications) so may have different trial populations.

Present the key data from these studies in a table: duration, population, region, phase, outcome measure, sample size, treatment effect, and other sample size parameters (e.g. variance, proportion in control group with success, median survival etc).

If there are anticipated differences between the chosen studies and the planned trial, it is important to understand how these impact on the assumptions used in the sample size calculation.

Recommendation: calculate an overall estimate (and confidence interval) of the treatment effect, population variability, median survival time etc., observed in previous studies using quantitative techniques such as meta-analysis.

The range of plausible values for the design assumptions can be used to assess how the sample size and / or statistical power changes over a range of assumptions (sensitivity analysis). The sample size under different assumptions can be tabulated, as shown below.

**Treatment Difference**	**Comparator response rate**				
	**22%**	**26%**	**30%**	**34%**	**38%**
**12%**	293	316	335	349	358
**14%**	219	235	248	258	264
**16%**	171	183	192	198	202

If researchers are uncertain about the reliability of their estimates, they may want to consider including a **sample size re-estimation procedure** in their study design.

Back to table

Sample size re-estimation

If there is uncertainty about the assumptions used in the sample size calculation, it may be prudent to check their validity using interim data from the study.

If researchers are revisiting the sample size during their study, they should include a description of the re-estimation procedure in the protocol.

Back to table

Report the Type I error

Type I error: false positive; rejecting the null hypothesis that is actually true in the population. Specify if the Type I error is **one or two sided**.

Back to table

Report the Type II error

Type II error: false negative; failing to reject the null hypothesis that is actually not true in the population.

Back to table

Adjustment for multiplicity

Adjustment for multiplicity may be required if there are: multiple outcome measures, multiple treatment arm comparisons, and multiple looks at accumulating data during interim monitoring (e.g. group sequential studies). Such multiple comparisons can increase both the Type I and the Type II error.

The strategy for controlling the Type I and the Type II error rates should be described. If researchers do not intend to adjust for multiple comparisons and / or looks then this should also be reported, together with an brief explanation of why they think adjustment is not necessary.

Back to table

The number of patients or events required for the analysis

The number of patients required for analysis is the **number obtained from the sample size calculation**.

Time-to-event study: the number of events required for analysis.

Back to table

Allowance for drop-outs

Drop-outs: include the expected proportion of participants with no post randomisation information (that is, participants lost to follow up).

Back to table

Total number of patients to be enrolled

Total sample size: total number of patients to be enrolled, i.e. after adjusting for drop-outs.

Back to table

Statistical Notes

Is it a representative sample?

If a study is to have value, we need to be confident that the sample studied represents the whole population (e.g. are the 4,000 patients in this high blood pressure study a fair sample of all people in the UK with high blood pressure?). If a sample is representative, the study results will be “generalizable”. It is therefore crucial that the inclusion and exclusion criteria, treatments, outcomes, and period of recruitment and follow-up are adequately described.

Back to table

Choosing the statistical test

There are various statistical tests available for analysing sets of data. Each of these procedures may be mathematically justifiable, but only if certain assumptions are satisfied about the data collected. These assumptions are embodied in the design for the study, which need to be explored and justified. It is important to involve a statistician from the beginning to ensure that the analysis plan is appropriate for the study.

There are basically two families of tests: parametric and nonparametric. Many statistical tests are based upon the assumption that the data are sampled from a normal or Gaussian distribution (see below). These tests are referred to as parametric tests. Commonly used parametric tests include the t-test and ANCOVA.

Tests that do not make assumptions about the population distribution are referred to as nonparametric- tests. Commonly used nonparametric tests include the Wilcoxon, Mann-Whitney test, and Kruskal-Wallis tests. These tests are also called distribution-free tests.

Caution: **If underlying assumptions are wrong, analysis may be nonsense.**

Does the outcome measure have a normal or Gaussian distribution?

The normal distribution is symmetric around the mean and is bell-shaped. The t-test is commonly used to determine whether the mean value of a continuous outcome variable in one group is statistically significantly different from that in another group. This test assumes that the distribution of the variables in each of the 2 groups is approximately normal (bell-shaped) and that the variances are equal.

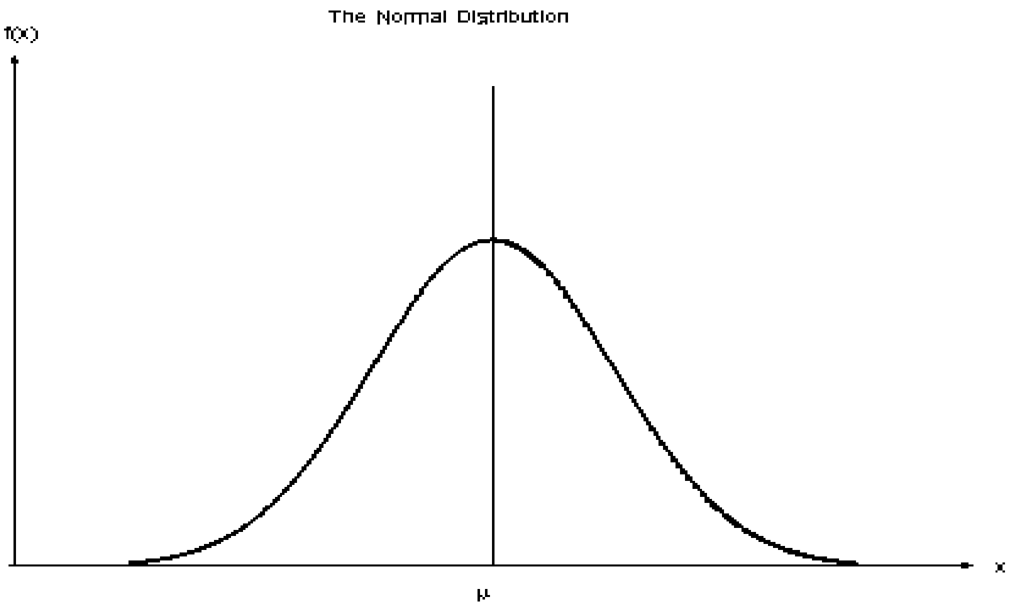

Back to table

Do the two groups being compared have the same degree of variability

Generally speaking, this assumption is a reasonable one to make. If this assumption is unlikely to be met then researchers need to base the sample size on a procedure that does not assume equal variability.

Back to table

Randomisation (or random allocation)

Random allocation is a means to ensure any confounding variables are distributed by chance between groups. In clinical research, statistical analysis is used to determine the reliability of any observed difference between two (or more) groups of participants.

If, as frequently applies, the purpose is to try to infer cause-and-effect (treatment A produces a different outcome to treatment B), we need to ensure the groups are similar as possible. The best way to achieve this is to allocate participants randomly (by chance alone). It doesn't guarantee that the two groups will be identical in terms of, say, age distribution or gender, or any other characteristic, but it does ensure that any differences between the groups should be small, and (most importantly) it enables limits on the degree of disparity between the groups to be calculated. This means that the degree of uncertainty in findings can be quantified. If people were allocated to groups by choice – if the clinician decided who was to be in which group – there is no way of even estimating how uncertain the study findings might be: in other words, the study would tell us nothing about the wider population that is of interest.

Back to table

Hypothesis testing, Type I and Type II errors, power and sample size

H0 – the null hypothesis – there is **no** difference between the two groups

H1- the alternative hypothesis - there **is** a difference between the two groups

There are two possible errors when a significance test is carried out. We can find an effect (accept H1) when in reality there is no effect (H0 is true); this is a **type I error**, and the probability of doing it is the significance level P. This would be a false positive. Or we can fail to find an effect (accept H0) when in fact there is one (H1 is true); this is a **type II error**. This would be a false negative.

False negatives can easily occur if the true effect is small, especially if there are relatively few data. If the researchers fix on a certain value to use for P (usually not greater than 5%), then the likelihood of a false negative occurring can be calculated for any specific size of true difference, and it depends only on the sample size (the amount of data). Researchers are therefore asked to justify their intended sample size by determining how likely it is that their study would detect a true difference of a specified size. They would normally be expected to specify what they considered to be the minimum clinically important difference, and then to calculate the sample size necessary to give a chosen probability (usually 80% or 90%) of giving a significant test result if that was the true difference. That probability (the 80% or 90%) is called the **power** of the test.

Just to emphasise: in a particular situation, with given outcome measures and hypotheses, once the P value is chosen, the power depends only on the sample size. The bigger the sample, the greater the power (nearer to 100%).

Note that too small a sample size may lead to a substantial risk that a worthwhile effect would go undetected by the researchers (an **underpowered study**). On the other hand, **too large a sample** when used for a significance test may risk finding a statistically significant effect (that is, we are fairly sure the effect is non-zero) when that effect is in fact too small to be of any value at all (we may be fairly sure it is non-zero but it may be too small to be worth bothering with).

Just to show how straightforward it is to **calculate the sample size**, let us consider an example with continuous data: suppose researchers wanted to compare two sets of participants given two different treatments for say blood pressure, and he or she judged that a difference of 5 mm mercury between the improvements brought about by the two treatments would be the minimum clinically important difference. Let's suppose the variability of improvements between individuals be measured by a standard deviation of 12mm, and suppose he or she used a P value of 5% and wanted a power of 90%. Then (subject to certain assumptions) the sample size for each group would need to be:

$n= 2 [(z_1+z_2) \times (\sigma/\delta)]^2$

where:

- n=sample size needed
- z_1 is a constant dependent on the desired P value. To reach a P value of 5% z_1=1.96
- z_2 is a constant dependent on the desired power. To reach a power of 90% z_2=1.28
- σ (sigma)=standard deviation of the measured variable
- δ (delta)= clinically relevant effect size (this is a clinical decision, not a statistical calculation)

in the case examined we would have:

$[(1.96 + 1.28) \times 12 / 5]^2 \times 2 = 120.9$, i.e. 121 per group

Back to top of document

References

1. Clark T. Ursula Berger, U. Mansmann, U. Sample size determinations in original study protocols for randomised clinical trials submitted to UK research ethics committees: review. *BMJ* 2013; 346:f1135
2. Altman DG. The scandal of poor medical research. *BMJ* 1994; 308:283.
3. Charles P, Giraudeau B, Dechartres A, Baron G, Ravaud P. Reporting of sample size calculation in randomised controlled trials: review. *BMJ* 2009; 338:b1732
4. Chan A-W et al: Discrepancies in sample size calculations and data analyses reported in randomised trials: comparison of publications with protocols. *BMJ* 2008; 337:a2299
5. Chan A-W, Tetzlaff JM, Gøtzsche PC, Altman DG, Mann H, Berlin J, Dickersin K, Hróbjartsson A, Schulz KF, Parulekar WR, Krleža-Jerić K, Laupacis A, Moher D. SPIRIT 2013 Explanation and Elaboration: Guidance for protocols of clinical trials. *BMJ* 2013;346:e7586.
6. National Institute for Health Research Reviewer Assessment Form: guidance for providing a review (professional contributors)
7. Wootton D. Bad Medicine: Doctors Doing Harm Since Hippocrates. *Oxford University Press*, 2006
8. Clarke M. Doing new research? Don't forget the old. *PLoS Med* 2004; 1:e35.
9. Evans I, Thornton H, Chalmers I, Glasziou P. Testing Treatments: Better Research for Better Healthcare. 2nd edition, *Pinter & Martin*, 2011.
10. ISIS-2 Collaborative Group. Randomised Trial of Intravenous Streptokinase, Oral Aspirin, Both, or Neither among 17187 Cases of Suspected Acute Myocardial Infarction. *The Lancet* 1988; 332: 349-360.
11. The DAMOCLES Study Group. A proposed charter for clinical trial 2005 data monitoring committees: helping them do their job well. *Lancet* 2005; 365: 711-22
12. EMA Guideline on Data Monitoring Committees. EMEA/CHMP/EWP/5872/03 Corr. London, 27 July 2005
13. FDA Guidance for Clinical Trial Sponsors: Establishment and Operation of Clinical Trial Data Monitoring Committees. March 2006
14. Chalmers I, Altman DG et al. Data sharing among data monitoring committees and responsibilities to patients and science. *Trials* 2013; 14:102
15. Schulz KF, Altman DG, Moher D, for the CONSORT Group. CONSORT 2010 Statement: updated guidelines for reporting parallel group randomised trials. *BMJ* 2010; 340:c332
16. Committee for Proprietary Medicinal Products (CPMP). Points to Consider on Switching between Superiority and Non-inferiority. CPMP/EWP/482/99. London, 27 July 2000
